# Drug Burden Index and its association with postural balance and falls among community-dwelling older adults

**DOI:** 10.3389/fmed.2026.1678855

**Published:** 2026-01-30

**Authors:** João Vitor H. Ribeiro, Marina L. A. Oliveira, Gustavo A. Gâmbaro, Rubens A. da Silva, Suzy Ngomo, Eros de Oliveira, Karen B. P. Fernandes

**Affiliations:** 1 School of Medicine, Pontifícia Universidade Católica do Paraná (PUCPR), Londrina, Brazil; 2 Graduate Program in Health Sciences (PPGCS), School of Medicine, Pontifícia Universidade Católica do Paraná (PUCPR), Curitiba, Brazil; 3 Center Intersectoriel en Santé Durable, Laboratoire de Recherche BioNR, Université du Québec à Chicoutimi (UQAC), Saguenay, QC, Canada; 4 Center Intégré de Santé et Services Sociaux du Saguenay–Lac-Saint-Jean (CIUSSS SLSJ), Specialized Geriatrics Services at La Baie Hospital, Saguenay, QC, Canada; 5 Center of Research in Health Sciences, University of Northern Paraná (UNOPAR), Londrina, Brazil

**Keywords:** Drug Burden Index, falls, medication burden, older adults, postural balance

## Abstract

**Background:**

Polypharmacy is linked to balance impairment and falls. Thus, pharmacological screening tools are essential for identifying high-risk patients.

**Objectives:**

To investigate the association between medication burden, postural balance, and recent falls in community-dwelling older adults.

**Methods:**

This cross-sectional study included adults aged ≥ 60 years attending a university outpatient clinic in Brazil. Sociodemographic characteristics, comorbidities, medication use, handgrip strength, and recent fall were collected. Medication burden was classified as no, low, or high according to the Drug Burden Index. Postural balance was assessed using a standardized one-legged stance protocol on a BIOMEC 400-412 force platform, with Center of Pressure (COP) area and sway velocity as primary and recent falls as secondary outcomes.

**Results:**

This study included 170 participants, and no demographic differences were found across DBI categories. DBI was significantly associated with postural balance, with greater COP area and sway velocity among individuals with low or high burden (*p* < 0.0001). In multivariable analysis, female sex was protective against balance deficit (OR = 0.35, 95% CI: 0.15–0.78, *p* = 0.01), and DBI demonstrated a strong dose–response pattern (low: OR = 7.29; high: OR = 30.66). Higher DBI also correlated with recent falls (trend χ^2^ = 5.91, *p* = 0.0001). ROC analysis of COP area resulted in an AUC of 0.75 (cut-off: 0.5; Accuracy: 72.9%).

**Conclusion:**

Higher DBI was associated with impaired balance and increased fall risk, with a 0.5 cut-off effectively identifying balance impairment and supporting DBI as a practical tool for detecting older adults at risk.

## Introduction

Falls are the most common cause of disability among older adults (1) and represent significant public health concern. It is estimated that nearly 30% of older individuals experience at least one fall each year, often leading to severe consequences such as fractures, increased dependency, institutionalization, reduced functionality, and even death (2). Assessing fall risk in older adults is complex due to its multifactorial nature (3). However, given that many falls are preventable, this issue warrants global attention as a critical area for intervention.

Polypharmacy is linked to balance impairment and a higher risk of falls in population-based studies (4–8). Evaluating static postural control in older adults may help identify potential balance problems and factors contributing to fall risk. Nonetheless, most of the existing evidence is derived from functional tests rather than objective measurements, such as Center of Pressure (COP) sway parameters. It is important to note that COP parameters obtained through force platforms enable a direct evaluation of balance deficits related to proprioception as well as postural adjustments, which encompass both feedback and feedforward mechanisms of the neuromuscular system. This enhances their reliability, as these aspects are usually limited in functional balance assessments (9).

In this context, the force platform is widely recognized as a valid instrument, providing quantitative and highly sensitive data for the analysis of postural control. A pioneering study by Goldie et al. investigated the reliability and validity of force platform measurements, and the results indicated that COP measures were the strongest predictors of postural stability, justifying the use of these parameters for identifying postural deficits and monitoring the progress of interventions (10).

Prescribing for older adults is particularly challenging, as treatment regimens are often complex and have a greater risk of polypharmacy (11). This, in turn, increases exposure to Potentially Inappropriate Medications (PIMs) and contributes to overall pharmacotherapeutic risk (12, 13). However, merely identifying polypharmacy may be insufficient, as some medications, especially those with sedative or anticholinergic effects, can be harmful even when used in monotherapy (14). Therefore, the use of pharmacological screening tools is essential for identifying high-risk patients and promoting safe and appropriate medication use.

The Drug Burden Index (DBI) quantitatively assesses the overall exposure to sedative and/or anticholinergic medications (15), and a high DBI has been associated with functional decline, falls, and several adverse outcomes in older populations (15–18). Considering that many psychotropic medications commonly used by geriatric patients have anticholinergic and sedative effects, the DBI may represent a more appropriate tool for population-based studies, as it provides a more comprehensive assessment of medicines with potential impact on both functional and cognitive performance.

While the DBI has been validated in various populations and settings (19), its validity has not yet been tested specifically in older adults from Latin America, such as Brazilians. Additionally, it has not been evaluated using objective measures of balance. Determining a potential cut-off point for assessing balance impairment in this population would be highly beneficial. This study aimed to investigate whether the medication burden, as measured by the Drug Burden Index (DBI), is associated with balance deficits and falls among physically independent older adults with preserved functional independence. Furthermore, the study aimed to establish a DBI cut-off point able to discriminate balance impairment in this population, thereby identifying a risk threshold for falls.

## Methods

### Ethical procedures

This study was approved by the local Research Ethics Committee (Irmandade da Santa Casa de Londrina Hospital Ethics Committee - named BIOISCAL, Protocol #: 36329214600000099). Before any procedures, all participants were informed about the nature of the study and were required to provide written, voluntary informed consent to confirm their agreement to participate.

In accordance with the Brazilian National Health Council Resolution No. 466/2012, participants did not receive any financial compensation for participating in the study. They were informed that their voluntary participation would contribute to research on medication safety and aging. After completing the assessments, participants received individual feedback on their balance performance, as well as general information on fall prevention strategies.

### Study design and sample size

Participants were recruited from the university medical outpatient clinic of Pontifícia Universidade Católica do Paraná (PUCPR), located in Londrina, Paraná State, Brazil. This clinic offers universal access to a variety of medical specialties within the public health system. Each year, approximately 13,000 patients are seen, with nearly 30% being older adults. For this study, we selected a convenience sample of older adults attending clinical appointments across different medical specialties.

Participants were community-dwelling older adults aged ≥ 60 years who were physically independent, defined as individuals able to perform basic and instrumental activities of daily living (ADL and IADL) without assistance. All participants were living independently in their own homes and did not require caregiver support for mobility, self-care, or household tasks.

#### Eligibility criteria

To be included in the study, participants had to meet the following criteria: any gender (as assigned at birth); be aged 60 years or older; provide voluntary consent; and sign the informed consent form.

Individuals with other known conditions related to balance impairment were excluded, such as full or partial prosthetics in any of the joints assessed; with a diagnosis of a musculoskeletal disorder, such as rheumatoid arthritis, systemic lupus erythematosus, or other rheumatic diseases; with post-traumatic or post-septic arthritis; with skeletal dysplasia; neurodegenerative disease; stroke; arrhythmias; chronic use of corticosteroids or immunosuppressive medications; heavy alcohol consumption; who had undergone arthroplasty, due to the inability to assess functional status. Additionally, exclusion criteria were extended to participants with hospitalization within the previous month or who refused to be involved in the research.

**FIGURE 1:**
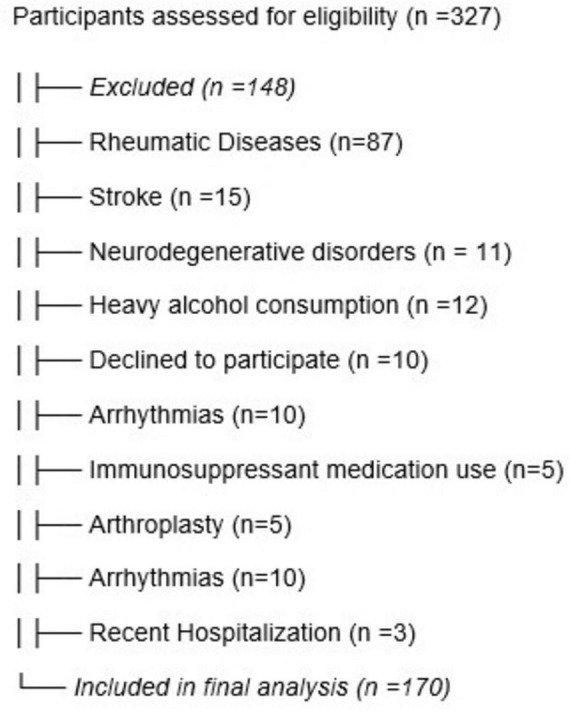
Flowchart of participant inclusion and exclusion.

A total of 327 community-dwelling older adults were screened for eligibility. After applying the inclusion and exclusion criteria, 173 participants met all requirements and were included in the analysis. The difference between screened and included participants resulted from the use of strict criteria to minimize confounding from pre-existing, mainly neurological, or musculoskeletal conditions and from functional dependence. These exclusions were intended to preserve internal validity and ensure that balance impairment reflected the pharmacological effects of medication burden. Figure 1 demonstrates a flowchart of the exclusion of participants.

### General assessment of the study population

A structured interview was conducted to collect socio-demographic data, regular physical activity, comorbidities, and medication use, as well as to characterize the profile of community-dwelling older adults.

Sociodemographic data (age, sex, race, education, and economic status), body mass index (BMI), comorbidity profile, and lifestyle (smoking and physical activity) were collected to be considered covariates in multivariable model.

The medication use was assessed through self-report, prescriptions, and chart review. All chronic pharmacotherapeutic schemes (conventional and herbal medicines) were recorded with their dosing regimens. Medication exposure was defined as the chronic use of prescribed medications for at least 6 months preceding the assessment, and their anticholinergic or sedative properties were quantified using the Drug Burden Index (DBI), as described by Hilmer et al. (15). Total drug burden was calculated by summing the individual DBI scores of each relevant medication. Medicines with both anticholinergic and sedative effects were classified as anticholinergics, in accordance with Kouladjian et al. (14). Based on DBI scores, participants were categorized into: No Burden (DBI = 0), Low Burden (0 < DBI < 1), High Burden (DBI ≥ 1), as recommended by Hilmer et al. (20).

### Blinding procedures

The assessor performing the balance tests was blinded to participants’ medication history and Drug Burden Index values. Medication data were extracted and coded by a separate researcher after the completion of balance assessments to minimize observer bias.

### Muscle strength and falls’ assessment

Handgrip strength was measured using a Jamar hydraulic hand dynamometer. A standardized protocol was strictly followed for data collection: participants were seated with their shoulders adjusted and in neutral rotation, elbows flexed at 90°, and wrists in a neutral position (21). Each participant was instructed to exert maximum grip strength for approximately 5 s. Two trials were performed for each hand, with a 30–60 s best interval between trials. The highest value recorded for each hand was used for subsequent analysis (21). The reference values described by Tomkinson et al. (22).

To assess recent fall occurrences, participants were asked how many falls they had experienced in the past 6 months. A fall was defined as “an unexpected event in which the person comes to rest on the ground, floor, or lower level” (23). A positive fall history was recorded if the participant self-reported a fall or if it was documented in electronic medical records.

#### Postural balance evaluation

Balance assessments were conducted by the same trained examiner using a BIOMEC 400-412^®^ force platform (BIOMEC 400-412, EMG System do Brasil Ltda, São José dos Campos-SP) to measure force reactions and linear Center of Pressure (COP) parameters during a postural control protocol. Participants performed a one-legged stance, barefoot, with arms at their sides and eyes open, following the protocol described by Da Silva et al. (24). In older adults, force platform assessments of postural sway under eyes-open conditions have shown excellent reliability, with intraclass correlation coefficients (ICC) above 0.80 for key center of pressure variables (25).

Postural control tasks were performed across two trials of 30 s each, with 60-s rest intervals between each trial to minimize fatigue during a standardized protocol. The participants were asked to stand barefoot, arms relaxed along the trunk, during the whole-time testing on a force platform, surrounded by a large wooden base (1.5 m × 1.5 m), facing a target (dot) on the wall, at eye level, 2 m from the platform, to stare at it during eyes-open tasks (26). Participant safety was ensured by a walking belt and a trained evaluator who was positioned near them during all data collection. Participants were recommended to be seated during recovery periods. The average across 2 trials was retained for subsequent analysis (26).

The sampling frequency was established at 100-Hz, and force signals were filtered with a 35-Hz low-pass second-order Butterworth filter and then converted into linear COP parameters, using the BIOMEC software combined with custom MATLAB software (The Mathworks Inc., Natick, Massachusetts) routines (27).

The 95% confidence ellipse area of the Center of Pressure (A-COP in cm^2^) represents the area that contains 95% of all COP positions during the trial. The primary balance parameters used for analysis were the mean COP ellipse area (cm^2^) and mean COP sway velocity (cm/s) in the anteroposterior and mediolateral directions, as previously tested in a Latin American population. Normal reference values were considered according to Oliveira et al. (28).

### Statistical analysis

Statistical analyses were performed using IBM SPSS Statistics version 20.0 and GraphPad Prism 5.0 for graph generation. A significant level of 5% (*p* < 0.05) and 95% confidence intervals were applied to all tests.

Following assessment of data normality with the Shapiro–Wilk test, the Kruskal–Wallis test with Dunn’s *post-hoc* test was used to compare medication burden across groups in relation to postural balance parameters. The *Chi*-square test for trend was employed to examine the association between the increase in medication burden and recent falls among the senior population.

To determine a potential cut-off point for drug burden associated with balance deficits–based on the Center of Pressure (COP) area–the receiver operating characteristic (ROC) curve analysis was conducted.

A multivariate model adjusting for key sociodemographic and clinical covariates was used. We modeled DBI categories against categorized main balance parameter (COP area) using Logistic Regression (Forward Stepwise Strategy) with robust variance to estimate Odds Ratios. The COP Area is dichotomized considering the cut-off point of 10.3 that is the reference value for balance impairment in Latin American population that can predicts falls (28).

Covariates include age, sex, Charlson Comorbidity Index, physical activity, and muscle strength. Firstly, we performed bivariate analysis, and only variables that were associated with the outcome (*p* < 0.20) were retained for the multivariate analysis.

## Results

**FIGURE 2:**
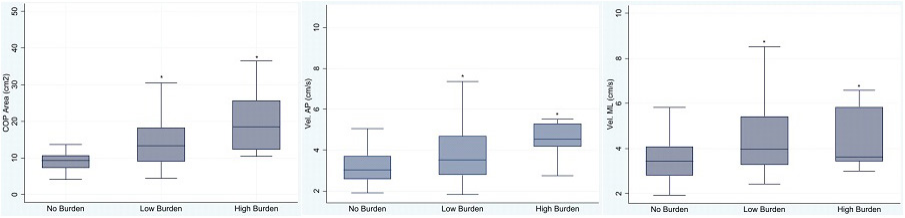
Association between medication burden (No Burden, Low Burden, and High Burden) and balance parameters: Center of Pressure area (COP), velocity in the anteroposterior direction (Vel. AP), and velocity in the mediolateral direction (Vel. ML). *Statistically significant, Kruskal–Wallis test, *p* < 0.05.

A total of 170 older adults were enrolled, and 124 (72.9%) were female. Table 1 presents the demographic features of the study population regarding medication burden. As no significant differences were observed among groups (Kruskal–Wallis test, *p* > 0.05), it can be stated that the groups were well matched with respect to general characteristics. Out of 170 participants, 101 (59.4%) experienced no significant medication burden, while 69 (40.6%) reported either low or high burden. Statistically significant differences were found in the Center of Pressure (COP) area (*p* < 0.0001), and in sway velocity in both the anteroposterior (*p* < 0.0005) and mediolateral (*p* < 0.001) directions with respect to DBI classification. Participants with low or high burden exhibited poorer balance parameters compared to those with no burden (Kruskal–Wallis test). The COP area proved to be the most reliable measure, effectively discriminating the three burden categories, as illustrated in Figure 2.

**TABLE 1:** General and clinical characteristics of the study population.

Variable	Medication Burden			*P*
	No Burden	Low Burden	High Burden	
**General characteristics and comorbidities status, median (1°. Q–3°. Q)**				
Age (years)	68.5 (64–74)	69 (63.7–74)	69 (65–75)	0.95
Weight (kg)	68.6 (58.2–77.1)	64.0 (56.7–74.8)	67.9 (58.5–78.0)	0.78
Height (m)	1.6 (1.5–1.7)	1.5 (1.5–1.6)	1.5 (1.5 -1.6)	0.99
BMI (Kg/m^2^)	27.1 (23.9–29.5)	25.8 (23.2–28.9)	27.5 (24.6–32.2)	0.22
Charlson Comorbidity Index	1 (1-2)	1 (1-2)	2 (1-2)	0.03
**Sex, *n* (%)**				
Male	29 (63.04)	10 (21.74)	7 (15.22)	0.04
Female	75 (56.39)	50 (37.79)	8 (6.00)	
**Regular physical activity, *n* (%)**				
No	48 (55.17)	32 (36.78)	7 (8.05)	
Yes	56 (60.87)	28 (30.43)	8 (8.70)	0.66
**Balance parameters, median (1°. Q–3°. Q)**				
COP area	9.36 (7.29–10.78)	13.23 (8.66–16.44)	18.86 (12.32–28.41)	0.0001
Vel. AP	3.07 (2.55–3.7)	3.47 (2.78–4.63)	4.79 (4.18–5.50)	0.0004
Vel. ML	3.42 (2.80–4.06)	3.98 (3.28–5.14)	3.64 (3.32–5.84)	0.0004

Kg, kilograms; m, meters; BMI, body mass index; AP, anteroposterior axis; ML, mediolateral axis.

In the bivariate analyses, female sex was associated with a significantly lower odds of balance deficit compared with males (OR = 0.38, 95% CI: 0.19–0.76; *p* = 0.006). After adjustment, this association remained significant, with women showing a 73% lower likelihood of balance deficit (OR = 0.27, 95% CI: 0.12–0.62; *p* = 0.002). Conversely, medication burden according to the Drug Burden Index (DBI) showed a strong dose–response relationship with balance deficit: individuals with low DBI had over six-fold higher odds (OR = 6.72, 95% CI: 3.15–14.31; *p* < 0.001), while those with high DBI had more than thirty-fold higher odds (OR = 33.81, 95% CI: 4.13–276.96; *p* = 0.001) compared to those without burden. Other factors, including age, Charlson Comorbidity Index, body mass index, physical activity, and muscle strength weakness, were not significantly associated with balance deficit in the multivariable model (Table 2). A statistical association was observed between recent falls and medication burden, with a higher prevalence of falls linked with a greater DBI (*Chi*-Square for trend = 5.91, *p* = 0.0001, Table 3).

**TABLE 2:** Logistic regression analyses of factors associated with balance deficit according to reference values of COP area described by Oliveira et al. (28).

Variable	Bivariate OR (95% CI)	*P*-value	Multivariable OR (95% CI)	*P*-value
Age range				
60–70 years	1.00		–	–
71–80 years	1.19 (0.55–2.57)	0.65	–	–
81–90 years	1.43 (0.32–6.40)	0.64	–	–
Sex				
Male	1.0		1.0	
Female	0.37 (0.16–0.84)	0.02	0.35 (0.15–0.78)	0.01
Charlson Comorbidity Index (per unit increase)	1.09 (0.86–1.39)	0.46	–	–
Regular physical activity				
No	1.0		–	–
Yes	1.04 (0.51–2.13)	0.91	–	–
Muscle strength weakness				
Yes	1.0			
No	0.49 (0.16–1.48)	0.20	0.54 (0.18–1.59)	0.26
Body mass index (per unit increase)	0.98 (0.92–1.05)	0.68	–	–
Medication Burden (DBI)				
No Burden	1.0		1.0	
Low Burden	6.98 (3.22–15.14)	0.0001	7.29 (3.39–15.68)	0.0001
High Burden	30.09 (3.58–252.90)	0.002	30.66 (3.69–254.81)	0.002

Model adjusted for age, sex, Charlson Comorbidity Index, handgrip weakness, physical activity, and medication burden according to DBI.

**TABLE 3:** Recent falls prevalence regarding medication burden according to Drug Burden Index (DBI) classification of the population study.

Medication Burden	Falls		Total
	No	Yes	
No Burden	67 (64.4%)	37 (35.6%)	104 (100.0%)
Low Burden	15 (25.0%)	45 (75.0%)	60 (100.0%)
High Burden	01 (5.5%)	14 (94.5%)	15 (100.0%)
Total	83 (46.4%)	96 (53.6%)	179 (100.0%)

Statistically significant, *Chi*-Square test for trend = 5.91, *p* = 0.0001.

**FIGURE 3:**
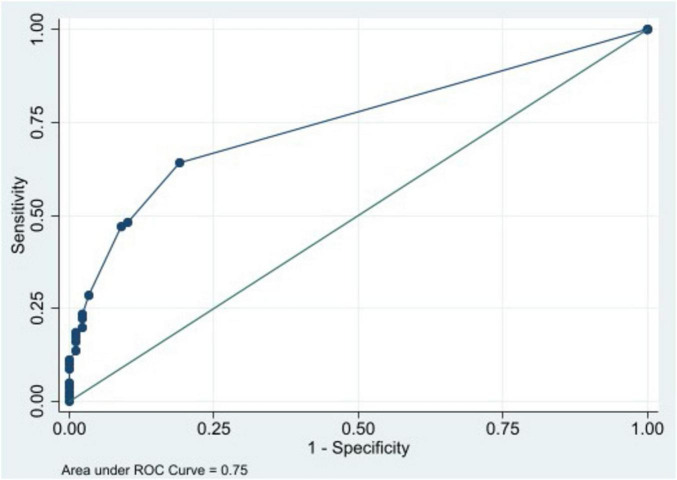
Receiver operating characteristic (ROC) curve representing the medication burden association with balance deficit, considering the Center of Pressure (COP) area parameter.

A ROC Curve was used to address the medication burden association with balance deficit, considering the Center of Pressure (COP) area parameter, and an area under the curve of 0.75 was determined, which indicates satisfactory performance. In this population, it was established that a DBI of 0.5 was the cut-off point to discriminate between individuals with and without balance impairment (Figure 3).

## Discussion

In this study, medication burden was associated with poorer postural balance and recent falls in older adults. These findings align with those of Clarkson et al. (29), who reported that higher drug burden is linked to increased hospital admissions due to falls. Similarly, Oya et al. (30) observed that individuals using multiple medications had a 20% higher risk of falling for each additional medication added.

Although polypharmacy is known to impair the physical and functional performance of older adults (31), evidence indicates that certain medications–particularly those with anticholinergic and sedative properties–may exert deleterious effects even when used individually (20, 32). This underscores the need for studies employing more refined and comprehensive tools capable of accurately capturing the complexity of pharmacological regimens, so that such approaches can be validated and integrated into clinical practice.

To our knowledge, this is the first study to examine the impact of medication burden on Center of Pressure (COP) parameters–considered the gold standard for assessing postural control–thus directly capturing balance deficits related to proprioception and postural adjustments across different population groups (33). In contrast, previous investigations in this area relied primarily on questionnaires, subjective scales, or functional tests, which generally demonstrate lower sensitivity (34).

In the multivariable analysis, female sex emerged as a protective factor against balance deficits, consistent with previous reports suggesting that women may exhibit distinct compensatory strategies or have lower exposure to certain high-risk medications compared with men. The protective association observed for women in our model may reflect sex-related differences in pharmacological exposure, physiological characteristics, or behavioral adaptations. Given the heterogeneity of the literature on sex differences in falls and medication-related risk, this finding should be interpreted with caution (35).

A notable strength of our study was the use of highly sensitive measures, which allowed us to detect balance differences across subgroups, including the distinction between low and high medication burden categories. Indeed, precise and direct outcome measures make a more meaningful contribution to clinical research and practice, as they enhance the ability to identify individuals at increased risk of falls (36).

Our findings highlight medication burden–quantified by the Drug Burden Index (DBI)–as an independent correlate of balance deficit, reinforcing previous evidence that cumulative exposure to anticholinergic and sedative medications compromises physical function and increases fall risk in older adults. The strength of the dose–response relationship we observed is consistent with cohort studies and systematic reviews demonstrating that higher DBI is associated with functional impairment and falls, supporting its use as a pragmatic tool for risk stratification across diverse clinical and population settings (19). Notably, the observed associations point more strongly to pharmacological class effects than to medication counts alone, aligning with evidence that sedative and anticholinergic load produces greater postural sway (37).

An important aspect of our results is that even low medication burden exerted a measurable negative impact on balance, with a cut-off value of 0.5 identified as sufficient to influence COP parameters in this population. This finding is particularly significant, as it underscores the need for early intervention among medicated older adults. Previous studies have generally classified individuals with high medication burden as the primary risk group (20, 29). The discrepancy may be partly attributable to the greater sensitivity of the outcome measures employed in the present study.

Unlike previous studies involving healthy community-dwelling older adults performing two-legged stance tasks, a further strength of our study is the use of a one-legged stance protocol–a more demanding postural control task that may be more predictive of balance impairment and, consequently, a stronger indicator of fall risk (38).

With respect to falls, we observed a trend indicating that greater medication burden is directly associated with a higher prevalence of recent falls, corroborating findings reported in other populations (6, 29). This reinforces the importance of clinical interventions in this context, as falls are linked to serious consequences, including fractures, institutionalization, reduced functional independence, and even mortality (30, 39).

Two key implications arise. First, incorporating DBI into routine medication review may help identify individuals at elevated risk even when functional testing is unavailable, aligning with proposals to adopt DBI as a practical screening tool (40). Second, our use of force-platform metrics (COP area and velocity) provides objective confirmation of balance impairment associated with medication burden, complementing prior research advocating for more sensitive instrumented measures beyond traditional functional assessments (41).

The use of indices capable of screening and discriminating high-risk individuals according to the deleterious effects of medications is particularly relevant, given that high-technology equipment is often unavailable in many clinical and population settings. In Australia, an electronic calculator incorporating the DBI formula has been developed and has demonstrated reliability and feasibility for bedside use (14). However, considering the variability in pharmacopeias across countries, this tool must be validated for diverse populations before broad implementation.

Several limitations warrant consideration. First, the cross-sectional design precludes causal inference, and prospective cohort studies are required to clarify temporal relationships. Residual confounding–such as unmeasured vestibular or sensory deficits–may remain, and medication exposure was quantified at a single time-point, even though dynamic fluctuations in medication burden can influence outcomes (42).

Alcohol use is a recognized confounder in studies examining medication burden and falls due to its potential interaction with sedative medications. Heavy drinkers were excluded during screening, reducing the likelihood of this bias. Additionally, alcohol intake was assessed through self-report, and no habitual heavy drinking was identified among participants. Nevertheless, self-reported measures are inherently susceptible to underestimation, and some degree of residual confounding cannot be completely ruled out. Future studies should incorporate objective or validated tools for assessing alcohol consumption to strengthen causal inference.

Although the exclusion of high-risk populations–such as individuals with Parkinson’s disease or cerebrovascular sequelae–may limit the generalizability of our findings, this methodological choice prioritized internal validity over external representativeness in this early phase of investigation. Future research should incorporate broader clinical populations with neurodegenerative or cardiovascular comorbidities and employ stratified or sensitive analyses to accommodate these conditions while preserving comparability.

In addition, the DBI quantifies cumulative pharmacological risk but does not provide a risk–benefit assessment for individual prescriptions, which represents an inherent limitation. A further constraint relates to the sampling procedure: participants were recruited from a single university-affiliated outpatient clinic, which may restrict the external validity of the findings. Consequently, the sample should be considered one of convenience, and caution is necessary when attempting to generalize the results to the wider older adult population. Nonetheless, the findings remain clinically relevant, as they offer real-world evidence from a context representative of patients who actively seek care within the public healthcare system. This data may help identify patterns of medication burden and fall risk in a population that is particularly vulnerable and highly dependent on public health services, underscoring the need for targeted interventions in similar settings. Indeed, replication in other Latin American populations is warranted.

Prospective studies that directly manipulate DBI–such as deprescribing trials–and monitor objective balance outcomes are required to determine whether reducing sedative and anticholinergic burden improves postural control and lowers fall risk. As DBI incorporates medication dosage and minimum recommended dose, it provides greater conceptual robustness compared with other indices (43). Evidence suggests that DBI may assist clinicians in identifying high-risk individuals who may benefit from adjustments to pharmacotherapeutic regimens or deprescribing interventions, with potential effects on reducing falls, frailty, and depressive symptoms (44, 45).

Thus, further research is needed to evaluate the generalizability of these findings across different populations, particularly considering the current absence of randomized clinical trials confirming the clinical effectiveness and safety of using DBI as a risk assessment tool in routine care.

## Conclusion

Medication burden, as quantified by the DBI, was independently associated with poorer postural balance and recent falls, even at relatively low levels of exposure. The DBI may function as a practical tool for identifying older adults at increased risk and informing safer medication management, although prospective studies are required to confirm its applicability and effectiveness across diverse populations.

## Data Availability

The raw data supporting the conclusions of this article will be made available by the authors, without undue reservation.
